# Guiding and monitoring focused ultrasound mediated blood–brain barrier opening in rats using power Doppler imaging and passive acoustic mapping

**DOI:** 10.1038/s41598-022-18328-z

**Published:** 2022-08-30

**Authors:** Aparna Singh, Jiro Kusunose, M. Anthony Phipps, Feng Wang, Li Min Chen, Charles F. Caskey

**Affiliations:** 1 Department of Biomedical Engineering, Vanderbilt University, Nashville, TN USA; 2 Department of Radiology and Radiological Sciences, Vanderbilt University Medical Center, Nashville, TN USA; 3 Vanderbilt University Institute of Imaging Science, Nashville, TN USA

**Keywords:** Engineering, Biomedical engineering, Drug delivery

## Abstract

The blood–brain barrier (BBB) prevents harmful toxins from entering brain but can also inhibit therapeutic molecules designed to treat neurodegenerative diseases. Focused ultrasound (FUS) combined with microbubbles can enhance permeability of BBB and is often performed under MRI guidance. We present an all-ultrasound system capable of targeting desired regions to open BBB with millimeter-scale accuracy in two dimensions based on Doppler images. We registered imaging coordinates to FUS coordinates with target registration error of 0.6 ± 0.3 mm and used the system to target microbubbles flowing in cellulose tube in two in vitro scenarios (agarose-embedded and through a rat skull), while receiving echoes on imaging transducer. We created passive acoustic maps from received echoes and found error between intended location in imaging plane and location of pixel with maximum intensity after passive acoustic maps reconstruction to be within 2 mm in 5/6 cases. We validated ultrasound-guided procedure in three in vivo rat brains by delivering MRI contrast agent to cortical regions of rat brains after BBB opening. Landmark-based registration of vascular maps created with MRI and Doppler ultrasound revealed BBB opening inside the intended focus with targeting accuracy within 1.5 mm. Combined use of power Doppler imaging with passive acoustic mapping demonstrates an ultrasound-based solution to guide focused ultrasound with high precision in rodents.

## Introduction

Focused ultrasound (FUS), when paired with microbubbles (MBs), can open blood brain barrier (BBB) reversibly and safely in animal models^1–4^. Opening BBB with FUS can help in successfully transporting various drug molecules such as doxorubicin and neurturin across BBB in small animals and can further enhance treatment of brain tumors and provide neuroprotection for Parkinson’s disease^5,6^. In clinical trials^7^, researchers have opened BBB using MR-guided focused ultrasound (MRgFUS) to deliver chemotherapy drugs to the brain in humans^7,8^ and demonstrated feasibility of BBB opening in patients with Alzheimer’s disease, Parkinson’s disease^9^ and amyotrophic lateral sclerosis^10^.

Focused ultrasound blood brain barrier opening (FUS-BBBO) typically targets specific regions of the brain and requires image guidance. In oncology applications, BBBO typically is targeted to the tumor region to enhance permeability to drugs or overcome the blood-tumor barrier^11,12^. In neuroscience applications, researchers have delivered molecules to elicit neuromodulation using schemes such as delivery of anesthetics^13,14^ or designer-receptors activated by designer drugs^15^ to specific brain regions of rodents. Accurate targeting is fundamental to experimental design of these studies, since the region of BBB opening must be located within a desired location (e.g., tumor, target brain region). Although MRI guidance is the gold standard and multiple systems exist that can apply FUS-BBBO under MRI guidance^16,17^ ultrasound guidance can also offer potential solutions by mapping anatomy, recording functional data, and monitoring therapy.

Ultrasound can also be used to monitor therapy by passively recording emissions during transcranial FUS procedures. Scientists have developed systems to map inertial and stable cavitation of microbubbles occurring during transcranial MRgFUS procedures via passively receiving emissions on an ultrasound array^18^ and have correlated spectral and spatial location of these echoes with BBB opened regions^19^. Another notable development that enhanced safety of FUS-BBBO procedures was a 3D subharmonic imaging technique that enabled BBB opening^20^ and predicted microbubble mediated tissue damage^21^. To accurately place the FUS focus in desired regions of the rodent brain outside of MRI, anatomical landmarks have been used^13^ while other researchers have used a cross grid placement on mouse skin of the skull which is visible in B-Mode image of an imaging transducer co-axially aligned to a single element FUS transducer^22^, achieving a targeting accuracy within 2 mm. In another study^23^, researchers targeted hippocampus within 0.5 mm of the actual FUS focus using single-element FUS transducer, with axial FWHM (= 13 mm) spanning entirety of the mice brain, to open BBB in mice using sutures of the skull as anatomical landmarks. Researchers, however, noted that sutures were not visible for all kinds of mice.

High frame rate Doppler imaging with microbubbles has recently established itself as a powerful neuroimaging tool capable of visualizing vascular structures and providing functional information in the brain^24,25^. Brain vasculature can be visualized by using ultrasound contrast agent during imaging procedures^26^. Using contrast agents, such as microbubbles (MBs), to create power doppler images revealed vasculature in the brain at a micrometer scale and made ultrasound localization microscopy and functional ultrasound possible^24,27,28^. By combining imaging of brain vasculature and functional activity with array-based steering, treatment planning with accuracy equivalent to MR-guided FUS procedures is possible.

Here, we developed methods to steer a FUS transducer array, with axial FWHM spanning only 2.7 mm, in a 2D plane using power Doppler imaging for guidance. We registered an imaging transducer with FUS array and used vascular reference points to guide the ultrasound focus to the intended target. During BBB opening, we received echoes at high frame rate to reconstruct passive acoustic maps using robust capon beamforming passive acoustic mapping technique (RCB-PAM)^29,30^ and overlayed these maps on power Doppler images^31^ to visualize the area undergoing cavitation. We quantified targeting error in our ultrasound-guided focused ultrasound (USgFUS) system in vitro using a cellulose tube in agar phantom and a cellulose tube in skull phantom with MBs flowing through it. The performance of power Doppler for image guidance was validated in three healthy rats by opening the BBB in a cortical region. BBB opening area was visualized using Gd-enhanced MRI and was compared to RCB passive acoustic map. By overlaying the FUS foci on the T_1_-weighted (T1W) MRI coronal slice, we visualized regions that underwent FUS treatment and BBB opening procedure. This study demonstrates an all-ultrasound method for combining power Doppler with a steerable focused ultrasound array to guide millimeter-scale FUS BBB opening while mapping and monitoring cavitation activity.

## Results

### Registering image and therapy coordinate systems

**Figure 1:**
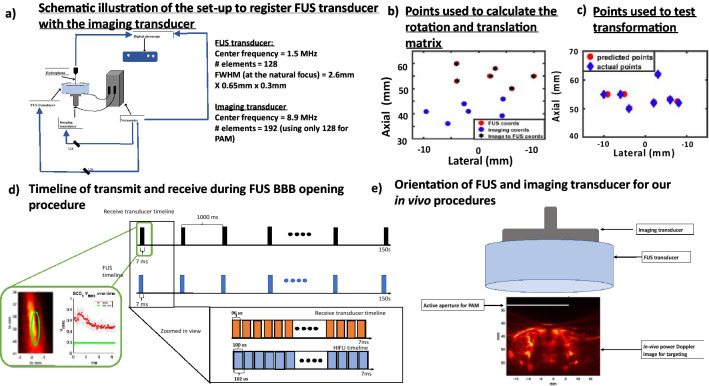
Registration of FUS transducer and FUS therapy. (**a**) Schematic diagram to record B-Mode hydrophone positions. Hydrophone positions were used to register the FUS transducer to the imaging transducer. (**b**) A total of 6 B-Mode images were collected to calculate rotation and translation matrix using Eq. 1, yielding a precise rigid transformation between the image and therapy space. (**c**) 7 target points were collected to verify registration and calculate target registration error (0.6 ± 0.3 mm). (**d**) The receive transducer recorded 96  µs for each 100  µs FUS pulse, which repeated at 9.8 kHz for a total duration of 7.0 ms during in vitro and in vivo applications. The in vitro application lasted 10 s and in vivo lasted 150 s. The receive signals were used to make passive acoustic maps and were used to calculate cavitation doses inside green box. (**e**) FUS transducer and imaging transducer were placed on top of rat skull. Imaging transducer was first used to collect power Doppler image at 50 Hz. This power Doppler image was then used for guiding FUS transducer. White band on power Doppler image shows active aperture of imaging transducer that was used to record receive echoes during FUS procedures.

Using experimental set up shown in Fig. 1a, we steered the FUS transducer to 6 points and positioned a hydrophone so that the pressure was maximized at each point. We then solved for registration transform between the hydrophone tip locations in the B-mode image (red circle in Supplementary Fig. S1a) and steering location in the FUS transducer coordinate system. We registered six imaging coordinates to six corresponding FUS coordinates to create a transform from imaging coordinates to FUS coordinates (Fig. 1b). The fiducial registration error (FRE) after registering these points was 0.18 mm. We then gathered a separate set of paired hydrophone and image steering locations and applied the transformation matrix to the imaging coordinates. The target registration error (TRE) between predicted points and actual points was 0.59 ± 0.26 mm (Fig. 1c). After confirming registration, we used our USgFUS system in in vivo and in vitro setting using sonication timeline in Fig. 1d. The receive signals were used to make passive acoustic map and were used to calculate cavitation doses.

### Power Doppler imaging with SVD filtering

**Figure 2:**
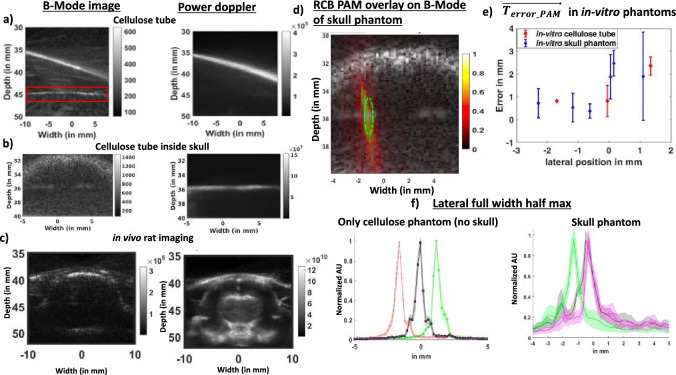
Targeting FUS with microbubble-enhanced power Doppler. (**a**) Using 8.9 MHz, SVD filtering of B-mode images removed stationary signals in the cellulose tube (red box highlights stationary boundary), and (**b**) similar SNR was feasible through the skull phantom. (**c**) In vivo imaging through the skull was performed at 5.2 MHz to account for skull thickness. (**d**) RCB passive acoustic map overlayed on skull phantom B-Mode image revealed regions undergoing cavitation in the skull phantom. Cavitation energy is contained within the green focus. (**e**) A $\overrightarrow {{T_{error\_PAM} }}$ comparison in in vitro cellulose tube and skull phantom, each undergoing 10 trials. (**f**) The point of maximum intensity in 3 steered maps was calculated and plotted in lateral dimension for cellulose phantom with and without skull.

After validating registration, we acquired high frame rate images of microbubbles under flow and applied an SVD filter to eliminate slow moving and static signal such as those seen at the boundaries inside red square in the B-Mode image in Fig. 2a to create power Doppler image in Fig. 2a. In an in vitro skull phantom, skull boundaries are present in B-Mode image in Fig. 2b. Power Doppler imaging in Fig. 2b removed skull boundaries but retained signal coming from MBs flowing through a tube inside the skull around depth of 36 mm. In vivo*,* power Doppler imaging could successfully differentiate between tissue and blood flow signals and removed slow moving signal from the B-Mode image (Fig. 2c) to create a transcranial vascular power Doppler image. Power Doppler imaging was successful in removing skull boundaries present in the B-Mode image and revealing vasculature underneath.

### RCB-PAM improves focal localization in the skull phantom

In deciding between implementing RCB-PAM or TEA-PAM, we reconstructed passive acoustic maps for entirety of FUS on pulse with both RCB-PAM and TEA-PAM algorithm in Supplementary Fig. S2 for our baseline and MBs data in presence and absence of skull. In our cellulose tube phantom case in Supplementary Fig. S2a, we saw that both algorithms could successfully create passive acoustic maps at targeted regions. However, TEA-PAM had tail artifact which was eliminated using RCB-PAM algorithm. In our skull phantom dataset in Supplementary Fig. S2b, we found that TEA -PAM could not create accurate passive acoustic maps. When we implemented RCB-PAM, however, we could resolve passive acoustic maps at target regions. For the remainder of this study, we only reconstructed our RF data with RCB-PAM algorithm and chose the first pulse because our preliminary analysis showed that, due to reperfusion every 1 s, first pulse contained the highest spectral content.

### RCB-PAM maps spatial location during steering

RCB passive acoustic maps reconstructed from signals received during FUS pulses designed for BBB opening exhibited greatest intensity inside of the ultrasound focus boundary (Fig. 2d and Supplementary Fig. S3). The focus had an intensity profile with similar spatial dimensions as the point spread function of the L12-5 array. Plot in Fig. 2f made from the location with maximum intensity across lateral dimension in Supplementary Fig. S3a for the 3 steering cases, each undergoing 10 trials, had a FWHM of 0.5 ± 0.1 mm, 0.5 ± 0.1 mm, and 0.6 ± 0.1 mm, while the intensity profile along the direction of the ellipsoid were 2.5 ± 0.2 mm, 3 ± 0.1 mm, and 2.5 ± 0.1 mm respectively. In the presence of skull shown in Supplementary Fig. S3b and Fig. 2d, RCB-PAM detected steering of the focus laterally and axially but resulted in wider FWHM in lateral and axial direction. Plotting out points, in Fig. 2f, of maximum intensity across the lateral dimension for 3 different steering cases in Supplementary Fig. S3b, with 10 trials in each case, resulted in FWHM of 0.8 ± 0.2 mm, 0.9 ± 0.1 mm, and 0.7 ± 0.2 mm while the steering profile along ellipsoid direction was 4 ± 1 mm, 4 ± 1 mm, 4 ± 1 mm respectively. RCB-PAM could localize signals in cellulose tube phantom in absence of skull and was used to calculate mean targeting error **(**$\overrightarrow {{{\varvec{T}}_{{{\varvec{error}}\_{\varvec{PAM}}}} }}$), the error vector between the pixel with highest energy and pixel that was the intended target. In the absence of skull, $\overrightarrow {{{\varvec{T}}_{{{\varvec{error}}\_{\varvec{PAM}}}} }}$ was 0.8 ± 0.7 mm, 0.8 ± 0.1 mm and 2.4 ± 0.4 mm across 10 pulses at each target with the largest error occurring when the focus was steered laterally beyond the extend of the active receive aperture on the L12-5 probe. When transmitting through the skull, RCB-PAM localized steering with sub-mm precision in three cases ($\overrightarrow {{{\varvec{T}}_{{{\varvec{error}}\_{\varvec{PAM}}}} }}$ of 0.4 ± 0.3 mm, 0.5 ± 0.6 mm, 0.7 ± 0.6 mm) and was over 1 mm in three cases (2 ± 1 mm, 2 ± 2 mm, and 2.5 ± 0.6 mm).

### Cavitation dose analysis in the presence and absence of skull

**Figure 3:**
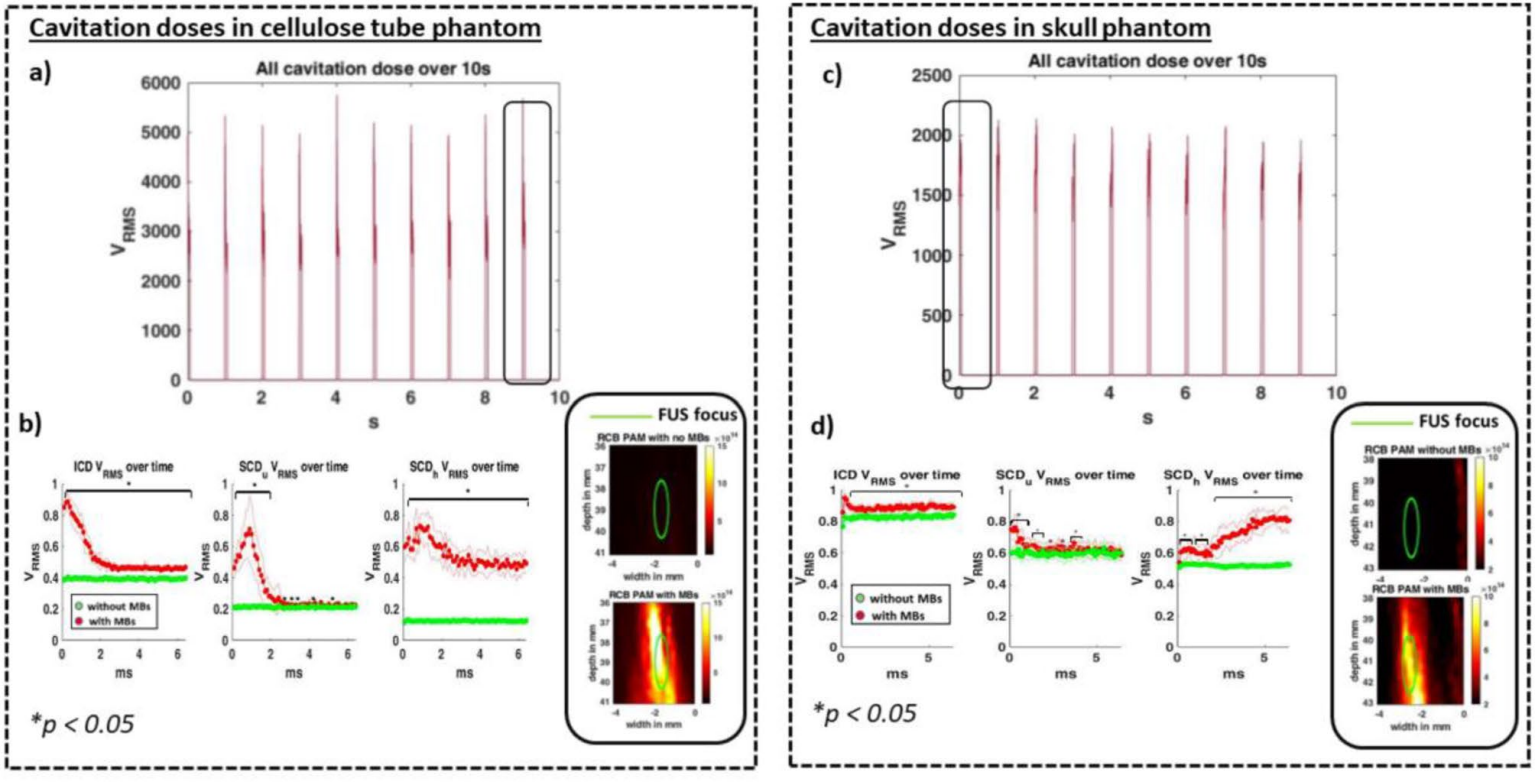
Cavitation dose analysis in in vitro phantoms. (**a**) Total cavitation doses (ie. the sum of ICD, SCDh, and SCDu) versus time in cellulose tube phantom. (**b**) During a single pulse, the mean ICD, SCDh and SCDu of each burst show that FUS sonication of MBs yielded greater doses than FUS sonications of saline and the doses were found to be statistically significantly greater. In ICD and SCDh, the values were statistically significant throughout the on pulse. The MBs peaks of ICD, SCDu and SCDh was 7.4 dB, 10.9 dB and 15 dB greater than saline respectively. A mean passive acoustic map of 69 pulses of the last 1 s sonication shows cavitation activity in the desired region in green circle. (**c**) Total cavitation doses (ie. the sum of ICD, SCDh, and SCDu) versus time in cellulose phantom through skull. (**d**) ICD, SCDu, and SCDh values during MBs sonications were found to be statistically significantly greater than saline sonications. The MB peaks of ICD, SCDu and SCDh was 1.6 dB, 2.5 dB and 4 dB greater than saline respectively. A mean passive acoustic map of 69 pulses of the fisr 1 s sonication shows cavitation activity in the desired regions.

In our in vitro cases, we performed separate set of experiments in presence and absence of skull where we kept FUS pressures of 0.6 MPa across both cases and flowed either saline or MBs diluted in saline through the cellulose tube. We found that total cavitation dose, which is sum of inertial cavitation dose (ICD), stable cavitation dose calculated from ultraharmonics (SCDu), and stable cavitation dose calculated from harmonics (SCDh), of MBs over 10 s in cellulose tube in Fig. 3a phantom was comparable across 10 s. Our cavitation dose analysis of only cellulose tube phantom in Fig. 3b revealed that mean ICD, and SCDh was statistically significantly higher when we sonicated MBs versus when we sonicated only saline (*p* < 0.05). In our SCDu analysis, we see that SCD was statistically significantly higher in MBs than in saline for first 3 ms and then went back to the baseline. The MBs peaks of ICD, SCDu and SCDh was 7.9 dB, 10.4 dB and 15 dB greater than saline respectively. A mean passive acoustic map of the last burst shows cavitation activity in the desired region in green circle. Similarly, total cavitation doses over 10 s in skull phantom is comparable in Fig. 3c. Cavitation dose analysis of our in vitro skull phantom in Fig. 3d showed the same trend where ICD and SCDh in presence of MBs were statistically significantly higher than in saline (*p* < 0.05). In SCDu, statistically significant results were only seen in the first 2 ms and sporadically thereafter. The MBs peaks of ICD, SCDu and SCDh was 1.6 dB, 2.5 dB and 4 dB greater than saline respectively. A mean passive acoustic map of the first pulse, made from 6.9 ms of RF data, shows cavitation activity in the desired region in green circle. We used ICD, SCDu, and SCDh values observed in skull phantom to compute thresholds over which values were considered statistically significantly greater in presence of MBs and were indicative of MBs activity (*p* < 0.05). These values were found to be 0.7 dB, 0.8 dB, and 0.76 dB for ICD, SCDu and SCDh respectively. We used these thresholds in our in vivo study to compute number of sonications that were indicative of MBs activity.

### Guiding FUS therapy with power Doppler and RCB-PAM feedback in in vivo rat brain

**Figure 4:**
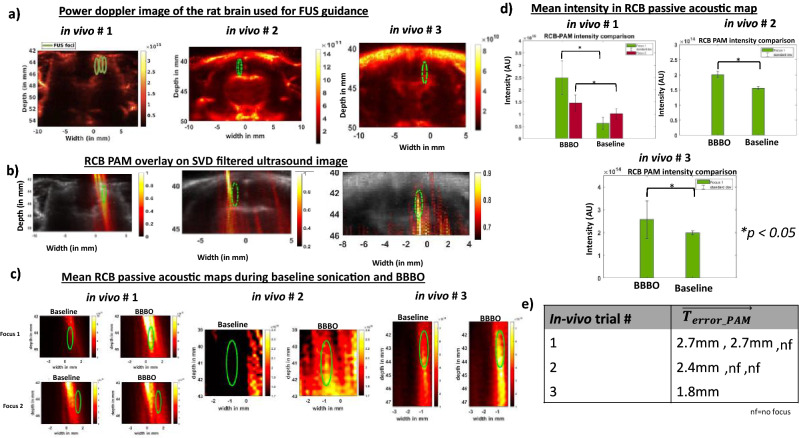
Power Doppler imaging can be used to guide BBB opening. (**a**) Power Doppler images from each in vivo case are shown with focal target overlaid in green. The first 2 trials have 3 foci and the third has a single focus applied as described in text. (**b**) RCB PAM, reconstructed from first 6.9 ms pulse just after MBs injection, overlayed on SVD filtered image show that PAM signals are present inside or near the intended FUS focus in first and third trials. (**c**) Cavitation maps show the relative difference between baseline and BBBO pulses during in vivo studies. (**d**) Mean RCB PAM intensity over 150 s reveal passive acoustic maps during BBBO have higher intensity than baseline maps for all maps that formed a focus in vivo. (**e**) Values of T_error PAM, computed from mean PAM maps constructed from first pulse of 150 s sonications for all three in vivo trials. Transmissions that did not form a focus on PAM maps (1/3 in trial 1 and 2/3 in trial 2 are denoted as nf).

The SVD doppler filtered power Doppler image revealed vasculature inside all in vivo rat brains (Fig. 4a). The vascular map served as a guide for FUS therapy as indicated by the green circle in Fig. 4a. We sonicated in vivo brains with 3 foci in trial 1 and 2, and with only 1 focus in trial 3. RCB passive acoustic maps, reconstructed from first 96 μs of sonication after of MBs injection, for all trials in Fig. 4b contain a majority of passive acoustic signal inside the SVD filtered brain image. We then reconstructed passive acoustic maps from the first 3 pulses of each 1 s sonication, i.e. 207 μs of 6.9 ms data, for both baseline and BBBO data (Fig. 4c and Supplementary Fig. S4a) in cases with 3 foci sonication and from the first pulse of each 1 s for single focus sonication.

We calculated mean intensity of all 150 BBB opening maps and compared it to mean baseline maps in Fig. 4c. In comparing maps, we see that BBB opened maps were higher in magnitude than baseline maps for all three in vivo trials. In our in vivo trial 1 in Fig. 4d, mean intensity of both foci were statistically significantly higher than baseline (*p* < 0.05). Focus 1 intensity was 12 dB greater in magnitude in presence of MBs. Spectrograms reconstructed from the last 30 μs in Supplementary Fig. S4b of our first pulse from our first sonication show in general more harmonic, ultra-harmonic, and inertial content in presence of MBs when compared to baseline sonication. Intensity of passive acoustic maps were also greater in our other two trials, and they were also found to be statistically significant (*p* < 0.05). $\overrightarrow {{{\varvec{T}}_{{{\varvec{error}}\_{\varvec{PAM}}}} }}$ for these mean passive acoustic maps in Fig. 4e were 2.3 mm and 2.7 mm for focus 1 and 2 for in vivo trial 1, 2.8 mm for in vivo trial 2, and 1.8 mm for in vivo trial 3. In our in vivo trial 1, RCB passive acoustic maps could only be seen in focus 1 and 2 whereas in in our in vivo trial 2, these maps could only be seen in focus 1.

### Cavitation dose analysis in vivo

**Figure 5:**
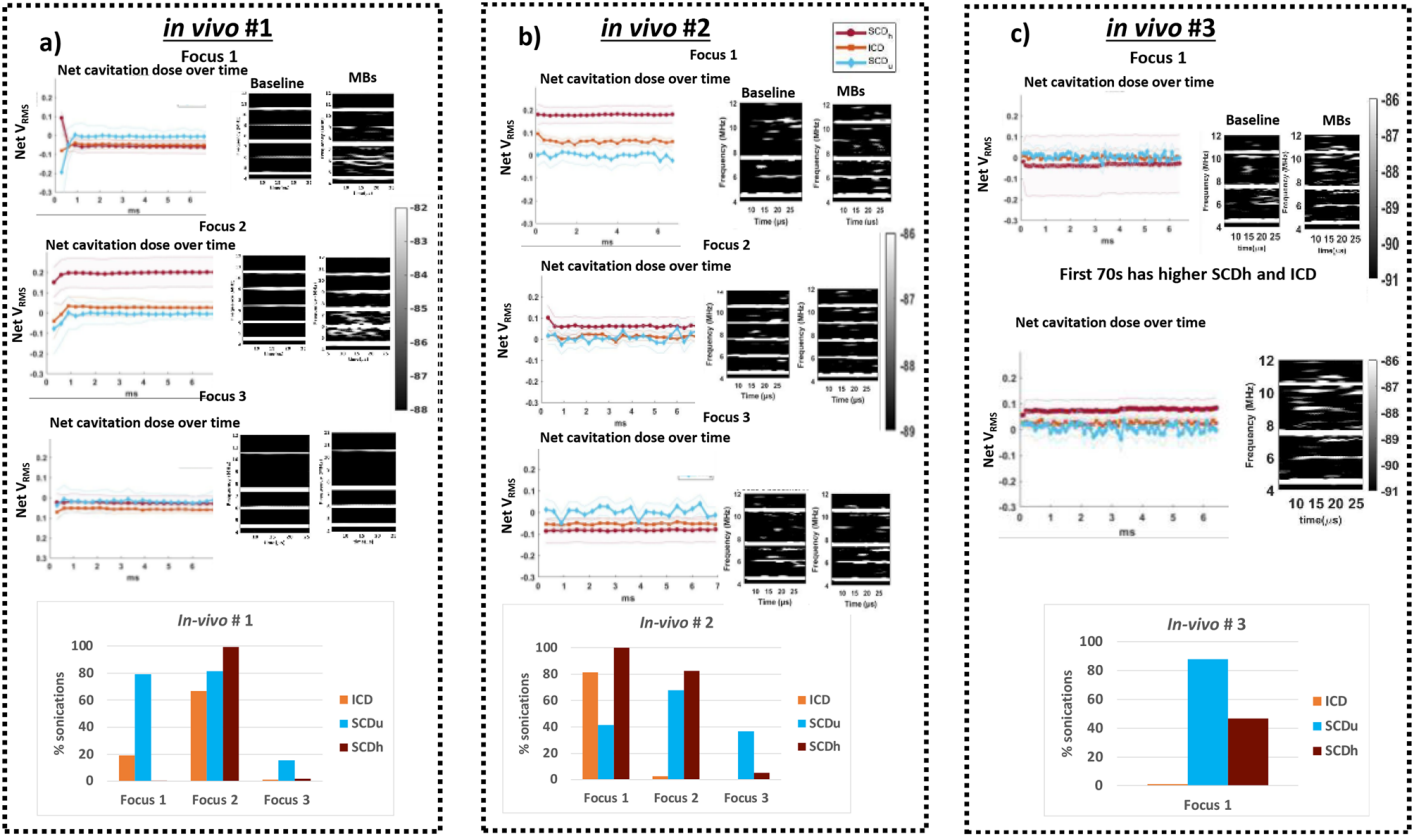
Cavitation dose analysis in our in vivo trials over 150 s of sonication. (**a**) In trial #1 where three foci were sequentially sonicated in a repeated manner, values of ICD and SCDh in focus 2 were greater after MBs administration, while focus 1 and focus 3 were comparable to baseline. Threshold analysis revealed that a total of 20% of sonications with focus 1 consisted of inertial activity and 66% consisted of ultra-harmonics activity that would be indicative of MBs activity (ie. at least 1 out of 23 pulses, and in some cases more than 1, resulted in inertial and ultra-harmonics activity which could be indicative of presence of MBs). (**b**) In trial #2, net values for ICD, SCDu and SCDh for focus 2 and focus 3, were near baseline, while echoes from focus 1 had greater values of ICD and SCDh after MBs administration. (**c**) In trial #3, we only sonicated using 1 focus and saw 88% of sonications above threshold in SCDu, 47% of sonications above SCDh threshold and, 1% of sonications above ICD threshold. Although average of 150 s sonication show doses comparable to baseline, in the first 70 s, SCDh and ICD were higher after MBs administration.

In our cavitation dose analysis of our first trial in Fig. 5a we saw that in focus 1 and focus 3, ICD, SCDu and SCDh were comparable to baseline as net V_RMS_ was close to 0. In focus 2, the values of ICD and SCDh were greater throughout after MBs administration. We used the threshold calculated in our in vitro experiment to identify number of sonications (out of 150) that had doses greater than threshold. Since each sonication was made of 69 pulses, we identified which of these 69 pulses consisted of values greater than threshold and if there was at least 1/69 pulse that was above threshold for ICD, SCDu, and SCDh, we considered this pulse, and subsequently sonication, to be indicative of MBs activity.

Threshold analysis in Fig. 5a revealed that a total of 20% of sonications with focus 1 consisted of inertial activity and 66% consisted of ultra-harmonics activity that would be indicative of MB activity. This means that at least 1 out of 23 pulses, and in some cases more than 1, resulted in inertial and ultra-harmonics activity which could be indicative of presence of MBs. This value was only 1% in SCDh case. In focus 2, 99% of sonications consisted of SCDh activity that would be indicative of MBs. Additionally, about 67% of sonications consisted of ICD activity above threshold and 81% of sonications consisted of SCDu activity above threshold. In focus 3, no such indications were observed in ICD, and less than 20% of sonications had any SCDu or SCDh values that would indicate presence of MBs. In our second trial in Fig. 5b, we saw that in focus 2 and focus 3, net ICD, and SCDu were comparable to baseline as the values were close to 0. In focus 1, the values of net ICD and SCDh were greater after MBs administration. Our threshold analysis revealed that a total of 100% of sonications with focus 1 consisted of some harmonics activity, 81% consisted of inertial activity, and 41% consisted of ultra-harmonics activity greater than threshold that would be indicative of MBs activity. In focus 2, about 83% of sonications consisted of SCDh and 68% sonication consisted of SCDu activity that would be indicative of MBs. Additionally, only 3% of sonications consisted of ICD activity above threshold. In focus 3, no such indications were observed in ICD, and less than 6% of sonications had any SCDh activity. However, 36% of sonications had SCDu activity greater than set threshold. In our third trials in Fig. 5c, we only sonicated using 1 focus. We saw 88% of sonications above threshold in SCDu, 47% of sonications above SCDh threshold and, 1% of sonications above ICD threshold. Although average of 150 s sonication show comparable doses to baseline with net cavitation dosage values close to 0, in the first 70 s, SCDh and ICD were higher after MBs administration.

### BBB opening confirmed via MRI

**Figure 6:**
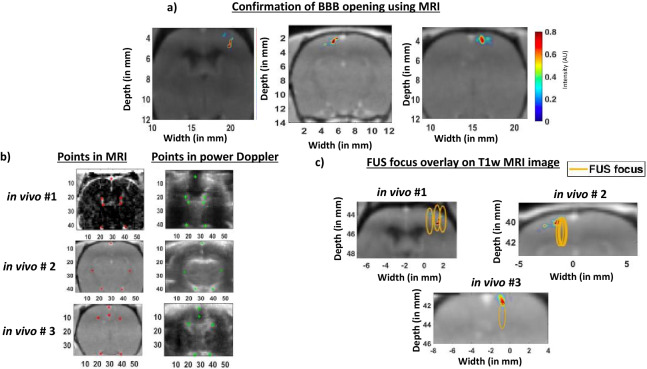
BBB opening was confirmed in MRI images and BBB opened regions overlaps with FUS focus. (**a**) BBB opening was confirmed in after post Gd-images. The colormap show the locations of BBB opened regions in all our in vivo trials. (**b**) Presence and detection of large ventricle and other features present in both power Doppler image and MRI image enabled in point registration of red stars in Gd subtracted MRI image was to green stars in power Doppler image. (**c**) After registering, FUS foci were overlayed on BBB opened MRI image. The BBB opened areas were inside the FUS focus in both in our fist two in vivo trials. In our third in vivo trial, the higher intensity of opening was seen inside the focus but majority of the opening was seen outside the FUS focus.

BBB opening in gadolinium images was visible in orthogonal planes (Fig. 6a). The volumetric BBB opening spanned 0.2 mm^3^ in in vivo trial 1 and 0.3 mm^3^ in both in vivo trials 2 and 3. The FWHM focal volume was 0.3 mm^3^. The BBB opening volume was less than total exposed volume of 0.96 mm^3^ (0.32 mm^3^ × 3 foci) in first two cases and the same as the focal volume in the third case. After registering (red points in Fig. 6b) MRI coordinates to anatomical landmarks in the SVD doppler image (green points in Fig. 6b), we overlayed intended FUS focus onto the rat brains (Fig. 6c) and observed the region of BBB opening co-localized with FUS focus. Using Eq. (10), we found that the targeting error, $\overrightarrow {{{\varvec{T}}_{{{\varvec{error}}\_{\varvec{BBB}}}} }}$ (the error vector between the pixel with maximum intensity in BBB opened region in the MRI image and the intended pixel in power Doppler image) was 1.1 for in vivo trial 1, 1 mm for in vivo trial 2, and 1.4 mm for in vivo trial 3.

## Discussion

In this study, we used MB-enhanced power Doppler images to steer a FUS transducer to open BBB in rats and performed retrospective analysis of echoes received to assess the ability to monitor regions undergoing therapy. We registered an imaging transducer with a FUS transducer and quantified the registration error. We characterized the ability of this system to target therapy and map bubble activity during MB-enhanced FUS therapy in an in vitro cellulose tube phantom with and without skull and found close correspondence between RCB passive acoustic map maxima and the steered FUS focal location. We applied these capabilities to open the BBB in rats and overlayed RCB passive acoustic maps and FUS foci on power Doppler image and Gd-subtracted T1W MRI respectively, demonstrating a 0.2 mm^3^ opening of BBB in vivo trial 1 and 0.3 mm^3^ opening of BBB in in vivo trials 2 and 3. The system provides a method to open the BBB in rats with high accuracy and spatial selectivity.

The choice of using the first 96 μs of data for single focus sonications and first 288 μs in multiple foci sonication data to reconstruct passive acoustic map is in line with other literature studies where it was found that 180 μs of data could accurately create in vivo passive acoustic map^19^ and 62.5 μs of integration time was found optimal for reconstruction of passive acoustic map from a 96.1 μs of RF data collected^32^. Our in vitro cellulose tube results without the skull in Supplementary Fig. S3 contained spectral energy in RCB passive acoustic maps within the FUS focus. The FWHM for most of the cases were in excellent agreement with FWHM of our focused ultrasound transducer. Only in one steering case, we saw axial FWHM 0.4 mm over the axial FWHM of FUS focus. The ${ }\overrightarrow {{{\varvec{T}}_{{{\varvec{error}}\_{\varvec{PAM}}}} }}$ for 3/6 cases was within sub-mm precision. In two cases however, the $\overrightarrow {{{\varvec{T}}_{{{\varvec{error}}\_{\varvec{PAM}}}} }}$ was over 2 mm. This $\overrightarrow {{{\varvec{T}}_{{{\varvec{error}}\_{\varvec{PAM}}}} }}$ was observed in lateral axis of around greater than 0.2 mm of the imaging transducer. We attribute the increase error in this region to only being able to receive on a sub-aperture, spanning the white band in Supplementary Fig. S1b and Fig. 1e (from − 18 to 6 mm), for our RCB-PAM sequence due to the way the 192-element array is addressed by our 128-element receiver. The reduced aperture would lead to an expected increase in the focal spot size achievable (on transmit and/or receive) at the edges of lateral extent of the receive. In all in vitro studies inside the skull, the FWHM was greater than FWHM observed in absence of skull. A study showed that presence of rat skull in the way of FUS beam path can distort and shift FUS focus by as much as 5 mm at 1.2 MHz^33^. Another study from our lab showed that both, steering a phased array and transmitting through the skull can result in an increase in axial FWHM^34^. Hence, a combination of steering the focus to the edge of the receive aperture and skull in the way FUS focus may be responsible for the higher FWHM and an average $\overrightarrow {{{\varvec{T}}_{{{\varvec{error}}\_{\varvec{PAM}}}} }}$ over 1 mm.

RCB-PAM overlay on power Doppler image of in vivo rat brains can reveal brain regions that underwent BBB opening. For our first in vivo BBB opening trial, the points we chose were 44.6, 44.3, and 44.4 mm axially and 0.6, 1.3, and 1.9 mm laterally for focus 1, focus 2 and focus 3 respectively. In our in vivo results, the spectral energy in the RCB passive acoustic map was within the brain in 2 out of 3 foci and the focus was lower and more diffuse in the case when energy was mapped outside of the brain. The passive acoustic map for 3 of the 7 foci in our in vivo study showed a diffuse focus with minimal activity inside the brain (Supplementary Fig. S4b), which we interpret to mean that a strong focus did not form at these points. The absence of foci in passive maps was associated with lack of wideband signals and increased amplitude and prevalence of harmonic content. The lack of a discernable focus on the passive acoustic maps combined with minimal detectable harmonic or wideband activity is consistent with the idea that a strong acoustic focus did not form, and we hypothesize that the combined effects of aberration and attenuation underlie this. The therapeutic array used in this work has a very low f-number^35^ so that many elements could have a higher angle of incidence that would not allow sound to transmit through the skull. Focusing schemes that consider aberrations in the path relative to each element would likely improve this performance^36^.In our in second in vivo trial, we did not see passive acoustic maps inside the brain for focus 2 and 3. In comparing dosage analysis in Fig. 5b, we found that focus 1 had mean SCDh and ICD for entire 150 s consistently higher than 0 throughout 6.9 ms of sonication. However, this was not seen for focus 2 and focus 3, which suggests that MBs may have fragmented by focus 2 and focus 3 since 1 MPa is well above inertial cavitation threshold of MBs.

Values of $\overrightarrow {{{\varvec{T}}_{{{\varvec{error}}\_{\varvec{PAM}}}} }}$ is higher in in vivo procedures which may be due to a combination of shift of FUS focus and attenuation of receive signal through the skull. The $\overrightarrow {{{\varvec{T}}_{{{\varvec{error}}\_{\varvec{PAM}}}} }}$ observed in our study was 2.7 mm for both focus 1 and 2 for in vivo trial 1, 2.4 mm for in vivo trial 2, and 1.8 mm for in vivo trial 3 in Fig. 4e.The $\overrightarrow {{{\varvec{T}}_{{{\varvec{error}}\_{\varvec{PAM}}}} }}$ values reported was less than 2 mm in 5/6 cases of skull in vitro. We attribute these higher $\overrightarrow {{{\varvec{T}}_{{{\varvec{error}}\_{\varvec{PAM}}}} }}$ to in our in vivo procedures due to skull (~ 1 mm) being thicker than our in vitro skull (< 1 mm) in trial 1 which could have resulted in attenuation of receive signal and may have also shifted the FUS focus as shown in one study^33^ where the focus shifted by as much as 5 mm at 1.2 MHz when a rat skull was introduced near the focus of a FUS beam. We believe that the signal was attenuated, especially in trial 1 because, although our intended target was at 44 mm axially, the point in the RCB-PAM images with the highest signal was located at 42 mm axially, which coincides with the location of the skull. Further MRI analysis confirmed that in fact the BBB opened regions were inside the brain away from the skull. Moreover, in our much simpler in vitro case, MBs was flowing through only one cellulose tube which was at FUS focus. Thus, MBs were flowing only at the FUS focus and not in FUS beam path. In our in vivo setting, MBs were present in vasculature throughout the brain and may have resulted in cavitation of MBs in the vasculature that are in FUS beam path in addition to cavitation at the FUS focus.

While our RCB-PAM algorithm worked well for controlled in vitro skull phantom analysis, it did not work as well for our in vivo analysis. There was lack of passive acoustic maps for different foci and while map intensities in presence of MBs were higher than in absence in trial 2, the passive acoustic map did not result in formation of focus. One of the reasons for lack of passive acoustic map could be lack of presence of broadband activity, which is indicated in Fig. 5b. Sonications of focus 1 produced SCDh in 100% of sonications and mean SCDh for entire 150 s reveal that the difference between SCDh produced during baseline and BBB opening was greater than when compared to difference between ICD produced during baseline versus BBB opening. Arvanitis et al. observed in their study that cavitation activity in passive acoustic mapping was more localized when they observed broadband and harmonic frequencies^19^. Another reason could be skull induced aberrations. Researchers have noted that if these skull-induced aberrations of acoustic emissions from microbubbles go uncorrected, it can introduce artifacts and image distortions in passive acoustic maps. To that end, researchers have applied aberration correction by combining information from skull CT with hemispherical array to improve accuracy of passive cavitation imaging^37,38^. In yet another study, researchers combined multi-modal imaging (ultrasound, MRI, and CT) with 3D finite difference time domain methods to account for spatially varying effects of ultrasound propagation and construct accurate passive acoustic maps in vivo^39^. In our in vivo experiment we did not implement any aberrations corrections, which may have resulted in lack of accurate passive acoustic maps. This issue can be overcome by applying aberration correction on both transmit and receive beamforming^36,38^, especially when transducers are centered at high frequency with a comparable focus size as ours^40,41^. Additionally, we can lower the frequency of our imaging transducer such that it is closer to resonance frequency of MBs. This will further improve accuracy of our PAM images and reduce $\overrightarrow {{{\varvec{T}}_{{{\varvec{error}}\_{\varvec{PAM}}}} }}$ values.

FUS focus overlay on Gd-Contrast MRI T1W MRI image of in vivo rat brain coincides with BBB opened regions and $\overrightarrow {{{\varvec{T}}_{{{\varvec{error}}\_\user2{BBB }}} }}$ show that our targeting is precise. However, there is a mismatch between volume of BBB opening and focal volume which could be due to lack of focus formation**.** We report $\overrightarrow {{T_{error\_BBB } }}$ values of 1.1 mm, 1 mm and 1.4 mm in the 2D plane where we applied and monitored therapy. These values are comparable to sub-millimeter accuracy of 0.9 mm ± 0.6 mm observed with 3D PAM^21^ and other studies with in vivo procedures^42–45^. Combining aberrations correction with ability to predict focal shifts can help us improve targeting accuracy and preserving focal volume shape. Prior work from our lab has shown that CT coupled with transcranial Kranion simulations can predict FUS focus shifts^46^. In our future studies, we plan to incorporate skull CT along with Kranion to inform ourselves of focal shifts and account for aberration corrections in both in transmit and receive end.

The observed BBB opening volume in our study ranged from 22% to greater than 100% of the volume exposed to FUS in our three animal studies. The focal opening percentage is within the range observed in a prior study in mice where BBB opening volumes approaching 100% of the FWHM were achieved in mice using comparable acoustic pulses^47^. Another study showed that using rapid acoustic short pulses yielded small BBB disruption which closed within 10 min^48^. Our opening volumes therefore align with others’ observations in rodents, but the variability of focal volume is higher than typically observed. Consistent and predictable BBB opening is a desirable goal for FUS therapy, and many factors underlie the degree of BBB opening: vasculature in treated brain region^49^, pulse duration^48,50^, microbubble dosage^50^, reduced focal pressure due to aberration^36^. It is likely that a combination of these factors contributed to the variability of the focal volume observed in our preliminary in vivo studies.

A smaller BBB opening enables excellent control over different regions of small animal brains. Of particular interest is drug delivery after FUS mediated BBB opening in small animals. Researchers have delivered chemogenetics to hippocampal regions under MR guidance^15^ in small animals. Our system can enhance the applications of such drug delivery by providing an all-ultrasound precise drug delivery to intended regions of brain with limited focal opening. The hippocampal regions in rat^51^ span around 70 mm^3^ and span around 20 mm^3^ in mice^52^. With the ability to generate a small acoustic focus, in conjunction with power Doppler imaging, our methods could open BBB in hippocampal regions with improved spatial selectivity. Another FUS application, neuromodulation, can benefit from our system where precise targeting of intended regions is of utmost importance. Previous studies showed that due to a smaller FUS focus at megahertz frequency, a successful motor activation of the limbs^53^, whiskers, and tail^54^ can occur with greater specificity. With a focal opening volume of up to 0.3 mm^3^ by our transducer, coupled with imaging, we can further enhance both specificity and spatial selectivity in the cerebral cortex regions of the rat and mice, which have volumes above 100 mm^3^^55,56^. Overall, we show that an all-ultrasound system can guide, steer, and target specific regions of the brain.

### Study limitations

Even with successfully performing BBBO under ultrasound image guidance, this study has some limitations. One limitation is that our system is only able to target for BBBO procedures within a 2D plane. BBBO in planes outside of the elevational focus of the linear array would likely not be detectable. Missed spatial information can be recovered by using 3D imaging arrays which many researchers have already used^25,57^ to map 3D functional activity in rodents. Using 3D imaging arrays, one can use power Doppler images to create 3D vascular images of the brain at different coronal planes and identify vasculature corresponding to specific areas of the brain. Coupled with already existing methods of constructing 3D passive acoustic maps^20,21^, one can steer electronic transducers in all three dimensions and target and record emissions originating in all dimensions with high accuracy.

A second limitation of our study is that we did not incorporate any feedback control during our BBB opening procedure. There are many approaches that have been implemented to open BBB safely in hundreds of animals and are based on monitoring specific frequency components such as harmonic^58^, subharmonic^59^, and ultra-harmonic^58,60,61^. Adding feedback control with cavitation dosages^62–64^ and investigating its effects on BBB opening and vasculature damage would likely enhance the application of our system. Additionally, the bandwidth of our transducer, which was between 5 and 12 MHz, may have posed additional limitations. MBs resonate around 1–5 MHz frequency^65^ and since we sonicated MBs at 1.5 MHz, we are uncertain that our receive transducer, which operated between 5 and 12 MHz, recorded all the echoes originating from MBs upon FUS sonication.

A third limitation of this study is that we only received on 128 elements of the 192-element array due to hardware limitations. The lateral extent of the imaging transducer, angular sensitivity, and directivity of elements determines the accuracy of RCB passive acoustic maps. While 2.4 cm of range gives enough coverage for most rodent brains, RCB passive acoustic maps beyond the lateral coordinates of imaging transducer have increased spatial inaccuracies. These spatial inaccuracies manifested in our in vitro data in the form of $\overrightarrow {{{\varvec{T}}_{{{\varvec{error}}\_{\varvec{PAM}}}} }}$ values greater than 2 mm (Fig. 2e) as foci were steered closer to the lateral extent of 6 mm of our imaging transducer.

The fourth limitation of the study is that RCB-PAM technique comes at a cost of high computational time. While RCB-PAM is very robust in removing interference and ‘tail’ artifacts that are otherwise present in TEA-PAM, it comes at a high computation cost^66^. Accelerating computation speed via a GPU can overcome this problem.

## Materials and methods

### Registering imaging transducer with focused ultrasound transducer

We used a 128-element focused (FWHM = 2.6 mm × 0.65 mm × 0.3 mm) ultrasound transducer^67^ with a center frequency of 1.5 MHz (Imasonic SAS, Besançon, France). This focused ultrasound transducer has an opening for the L12-5 imaging transducer such that it is coaxially aligned with the FUS transducer. We sonicated a 10-cycle FUS pressure pulses at 1.6 V (0.5 MPa) input voltage with a pulse repetition frequency of 1.5 kHz using Verasonics Vantage system (Fig. 1) (Verasonics Inc,Kirkland, WA, USA). We steered the focus to a location within the image plane and positioned the hydrophone using a manual 3-axis manipulator. A digital oscilloscope (Picoscope software, Pico technology, Tyler, TX, USA) was used to display the signal recorded by needle hydrophone (H0200, ONDA, Sunnyvale, California, USA) and displayed the signal on a computer. We maximized this signal so that the hydrophone tip was located at the focus. To detect the location of sonication in the image space, we set the L12-5 transducer in B-Mode at 8.9 MHz using Verasonics and recorded the location of the hydrophone in the image. We saved the IQ data for post-processing to find the pixel with maximum intensity which would correspond to the tip of the hydrophone as shown in Supplementary Fig. S1a. This procedure was repeated for 6 different locations to determine a registration between the imaging and therapy transducers. After collecting the points, we computed the rotation matrix and translation vector required for rigid transformation using SVD solution, described elsewhere^68^, such that Eq. (1) was satisfied^69^

1 $$\varvec{y}_{{\varvec{FUS}}} \varvec{'} = R\varvec{x}_{{\varvec{imaging}}} + \varvec{t}$$

We estimated our fiducial registration error using these transformed 6 points in Fig. 1c. After calculating rotation and translation matrix, we gathered 7 more points at imaging and FUS coordinates to compute the target registration error of points that were not used as input fiducials. We transformed these 7 imaging coordinates into FUS coordinates and compared transformed coordinate values (red circles) with ground truth FUS coordinates (blue diamond) in Fig. 1d. Using transformed points and actual points, we calculated target registration error.

### Microbubble fabrication

We produced lipid-shelled MBs in-house following the methods described in Borden et al. 2005^70^. Briefly, we combined and dried into film 90 mol% 1,2-distearoyl-sn-glycero-3-phosphocholine (DSPC) and 10 mol% 1,2-distearoyl-sn-glycero-3-phosphoethanolamine-N-[amino(polyethylene glycol)-2000] (DSPE-PEG2k) and resuspended it to 2.5 mg/ml in MB buffer solution (80% v/v of 0.9% NaCl, 10% propylene glycol [1,2-propanediol], and 10% glycerol). We degassed the solution and filled the head space with F10C4 (FluoroMed, Round Rock, TN, USA). We repeated this process for a total of 3 times. We agitated bubble solution using a VialMix (DuPont, Wilmington, DE, USA) for 45 s to form MBs prior to experimentation. We purchased DSPC and DSPE-PEG2k from Avanti Polar lipids (Alabaster, AL, USA).

### Confirmation of registration by using rotation and translation matrix in in vitro phantoms of cellulose tube with and without skull phantom surrounding it

We first tested our registration in a 200 µm cellulose tube (Spectra/Por, Spectrum Laboratories Inc., USA) embedded in 2% agar (NOW Foods, IL, USA) phantom and then in cellulose tube inside a skull phantom. The concentration of MBs and flow rates were identical in both in vitro phantom. Briefly, we diluted MBs in saline at a concentration of 50 µL in 1 mL to ensure there were enough MBs present for all trials. While the MBs were flowing in cellulose tube at 12 mm/sec, we captured and saved B-mode images at 50 Hz. We captured a total of 1000 frames at 8.9 MHz. We subjected these 1000 frames to an SVD filter to eliminate any static signals, thereby leaving only MBs signals^31^. We selected a cutoff of 250 singular values to filter any static signal. After we applied an SVD filter, we displayed SVD reconstructed image was on the screen and used MATLAB’s ginput function to select target points on the cellulose tube. We used rotation and translation matrix to transform this selected point into FUS coordinates system and calculated amplitude and phases of the FUS transducer to steer to this point^71^. We sonicated this point with a 100 µs pulse at 1.5 MHz with 1.5 MPa free field pressure in the cellulose tube and at 2.5 MPa free field for cellulose tube in the skull phantom. While the FUS was on, we set the first 128 elements of imaging transducer in receive mode to receive the first 96 µs of receive data for each 100 µs of FUS on time. The active aperture for RCB-PAM is shown in the wideband in Supplementary Fig. S1b and Fig. 1e. We sonicated with a total of 67 bursts of 100 µs pulses every 1 s. We repeated this transmit on FUS and receive on imaging for a total of 10 times at 1 Hz PRF for one point and repeated this procedure for different steered position for the in vitro phantoms (i.e., three steered positions for agarose-embedded tube and six different steered positions for tube within skull). We processed the receive data offline to reconstruct passive acoustic map using robust capon beamforming method passive acoustic mapping (RCB-PAM) due to its ability to improve spatial resolution and removal of any incoherent artifacts, especially in presence of a skull, as shown in Supplementary Fig. S2^29^. The RCB-PAM algorithm can localize acoustic cavitation activity^72^ and is briefly explained below. Assuming that there is a single acoustic event at location r with acoustic strength $q\left( {{\varvec{r}},t} \right)$, the pressure generated at point $\user2{r^{\prime}}$ at time $t$ will be:

2 $$p\left( {\user2{r^{\prime}},t} \right) = \frac{{q\left( {t - \frac{{\left| {\user2{r^{\prime}} - {\varvec{r}}} \right|}}{c}} \right)}}{{4\pi \left| {\user2{r^{\prime}} - {\varvec{r}}} \right|}}$$

where *c* is the speed of sound in the medium. We set the speed of sound for phantoms in this study between 1440 and 1460 mm/s to account for refrigeration of our skull and cellulose tube phantom at 4 °C^73,74^ and at 1480 mm/s for water at 20 °C for our in vivo study. We refrigerated this phantom to prevent denaturation of the phantom and to ensure repeatability of our experiment. Equation 2 considers the spherical propagation of the acoustic wave and time of arrival from the source of observer. By inverting Eq. 2, we can estimate acoustic source strength based on the acoustic pressures $\tilde{p}({\varvec{r}}_{i} ,t)$ detected at each pressure sensor ${\varvec{r}}_{{\varvec{i}}}$ for course of time $t$. The estimated acoustic strength $\tilde{q}({\varvec{r}}_{i} ,t)$ is then calculated by applying relative delays to the acoustic signal of the $i$-th channel and averaging it across the *N* channels:

3 $$\tilde{q}\left( {{\varvec{r}},t} \right) = \frac{1}{N}\mathop \sum \limits_{i = 1}^{N} 4\pi \left| {{\varvec{r}}_{{\varvec{i}}} - {\varvec{r}}} \right|w_{i} \tilde{p}\left( {{\varvec{r}}_{{\varvec{i}}} ,t + \frac{{\left| {{\varvec{r}}_{{\varvec{i}}} - {\varvec{r}}} \right|}}{c}} \right)$$

where $w_{i}$ is the weight applied to the $i$-th channel. The RCB method determines the apodization weights $w_{i}$ appearing in Eq. (3) that helps with suppression of interference pattern that otherwise arises in TEA–PAM algorithm. Eventually, we can use $\tilde{q}\left( {{\varvec{r}},t} \right)$ to calculate estimated acoustic energy radiated by a single acoustic event using Eq. (4) below:

4 $$\tilde{E}\left( {\varvec{r}} \right) = \user2{ }\frac{1}{{4\pi \rho_{0} c}}\mathop \smallint \limits_{0}^{T} \widetilde{{q^{2} }}\left( {{\varvec{r}},t} \right)dt$$

where $\rho_{0}$ is the density of the medium and *T* is the total duration of the signal.

In matrix format, Eq. 3 can be written as:

5 $$q\left( {x,t} \right) = \frac{4\pi }{\alpha }{\varvec{w}}^{{\varvec{T}}} {\varvec{D}}\left( {\varvec{x}} \right){\varvec{s}}\left( {{\varvec{x}},t} \right)$$

where $\alpha$ is the piezoelectric coefficient, ${\varvec{w}}^{{\varvec{T}}}$ is weighting matrix applied to each channel, ${\varvec{D}}\left( {\varvec{x}} \right)$ is the diagonal matrix of distances from channel 1 to N to pixel ${\varvec{x}}$ of interest:

6 $${\varvec{D}}\left( {\varvec{x}} \right) = diag\left[ {d_{1} \left( {\varvec{x}} \right), \ldots d_{N} \left( {\varvec{x}} \right)} \right]$$

and ${\varvec{s}}\left( {{\varvec{x}},{\varvec{t}}} \right)$) is matrix of pre-steered data of N channels. A correlation matrix of pre-steered for an interval length of T can be written as:

7 $${\varvec{R}}_{{\varvec{s}}} \left( {\varvec{x}} \right) = \mathop \smallint \limits_{{t_{0} }}^{{t_{0} + T}} {\varvec{s}}\left( {{\varvec{x}},t} \right){\varvec{s}}\left( {{\varvec{x}},t} \right)^{{\varvec{T}}} dt$$

The source energy, for each pixel is then:

8 $$E\left( {\varvec{x}} \right) = \frac{4\pi }{{\alpha^{2} \rho_{o} c}}{\varvec{w}}^{{\varvec{T}}} {\varvec{D}}\left( {\varvec{x}} \right){\varvec{R}}_{{\varvec{s}}} \left( {\varvec{x}} \right){\varvec{D}}\left( {\varvec{x}} \right){\varvec{w}}.$$

The value of T was set to 96 µs. We beamformed 1/67 bursts of 100 µs of data and used bursts that resulted in an RCB PAM for further analysis. RCB PAM was computationally expensive, requiring approximately 5 h to compute maps for a 10-trial data set. Dimensions of 7 mm × 8 mm does not cover the entire imaging plane and only covers a subset. Increasing dimensions to cover entire imaging plane would add computational time. Therefore, we chose to use the first pulse, which is also the pulse with highest spectral intensity according to our preliminary analysis. We estimated the 2D spatial location of maximum intensity in the that passive acoustic maps ($pixel_{PAM} = \left[ {x_{PAM} ,y_{PAM} } \right])$ and the location of the pixel that was clicked on the power Doppler image ($pixel_{PD} = \left[ {x_{PD} ,y_{PD} } \right]$) to calculate targeting error vector ($\overrightarrow {{{\varvec{T}}_{{{\varvec{error}}\_{\varvec{PAM}}}} }} )$ using Eq. 9:

9 $$\overrightarrow {{{\varvec{T}}_{{{\varvec{error}}\_{\varvec{PAM}}}} }} = \sqrt {(x_{PAM} - x_{PD} )^{2} + (y_{PAM} - y_{PD} )^{2} }$$

We normalized passive acoustic maps to it maximum intensity only to overlay on B-Mode images. In a separate set of experiments, we sonicated MBs flowing in cellulose at 0.6 MPa in presence and absence of skull and also collected baseline control data by sonicating our negative control, saline. We, then, compared cavitation doses in presence and absence of skull, with and without MBs.

### Spectrogram visualization

We collected the receive RF data at sampling frequency of 35.6 MHz used this data to compute spectrograms. For the spectrograms, a total of 400 samples were overlapped and total of 404 points were used to compute discrete Fourier transform. We grouped spectrograms from baseline FUS sonication and MBs assisted FUS sonication to visualize harmonic content (i.e. *f*_*n*_ = n*f_0_, *n* = [1,2,…8]) and ultra-harmonic content (i.e. *f*_*n*_ = (*n* + 1/2)*f*_0_, *n* = [1,2,…8]) in Fig. 3*.* Furthermore, we used these spectrograms and filtered ± 150 kHz range at transducer’s harmonic, and ultra-harmonic content to calculate inertial cavitation dose, stable cavitation dose from harmonic content of receive signals, and stable cavitation dose from ultra-harmonic content of receive signals using root mean square method^62^. Spectrogram processing was applied to all received echoes. After calculating cavitation doses, in presence and absence of MBs, we normalized ICD, SCDh, SCDu of all cases to maximum ICD, SCDh, and SCDu in MBs respectively. For our in vitro analysis, we presented these normalized doses in Fig. 3. For our in vivo case, we subtracted mean ICD, SCD, and SCDu doses in absence of MBs from doses in presence in MBs to present net cavitation dose (i.e. Net V_RMS_) in Fig. 5.

### FUS blood brain barrier opening in vivo

All procedures were reviewed and approved by the Vanderbilt University Institutional Animal Care and Use Committee and performed in accordance with the relevant guidelines and regulations for animal research. Additionally, our study was in compliance with the ARRIVE guidelines. We anesthetized three male Sprague–Dawley rats (250–568 g) using isofluorane and performed catheterization to enable MBs injection. We shaved the head and used Nair (Nair Hair Remover Lotion, Church & Dwight Co., USA) lotion to remove hair to improve coupling. Ultrasound gel was applied and the FUS and imaging transducer were placed on the rat head as shown in Fig. 1e. We injected MBs in rats and used L12-5, to capture, reconstruct and save 1000 frames at frame rate of 50 Hz at 5.2 MHz. Each frame was composed of 7 compounded planes waves ranging from − 18° to 18°. We applied an SVD filter according to steps listed above and displayed the SVD filtered image on the screen. We used MATLAB’s ginput function to select sonication points in the brain and transformed them into FUS coordinates system using the rotation and translation matrix. We sonicated 3 foci in first two in vivo trials and with one focus in third in vivo trial. We calculated amplitude and phases of FUS transducer using methods described elsewhere to steer to these points^71,75,76^. We sonicated multiple foci sequentially with 69 pulses where each pulse was 100 µs and was repeated at 9.8 kHz with a center frequency of 1.5 MHz and peak negative pressure of 0.8 MPa in in vivo trial 1, 1.1 MPa in vivo trial 2 (ie. 7 ms sonication with 98.57% duty cycle). The 6.9 ms of transmitted sound was distributed amongst 3 points so that each point was sonicated for a total of 2.3 ms every second. In the single focus sonication in trial 3, we sonicated the same focus for entirety of FUS pulse at 0.7 MPa. These pressure values were derated from free-field measurements due to skull properties and weight of rat^77^. We coupled our imaging and FUS transducer and used Doppler imaging to localize our position on the skull. Using linear equation at transmission frequency of 1.5 MHz described in paper^77^, we estimated that only 25% of free-field pressure transmission occurred in rat 1, whereas about 60% of free-field transmission happened in rats 2 and 3. These 69 pulses were repeated for a total of 150 times at 1 Hz PRF accounting for 150 s of treatment duration as shown in Fig. 1d. Before collecting BBBO data, we also collected 10 s of baseline data where we sonicated the desired regions but before MBs injection. We accounted for the weight of the animal, position of our focus on FUS transducer, and FUS transmission frequency to calculate pressure transmission factor^77^. While the FUS was on, we received the first 96 µs of RF data for each 100 µs of FUS on time on L12-5. We performed this transmit on FUS and receive on imaging for a total of 150 s and collected receive data for all 150 s. We processed the receive data offline to reconstruct PAM using RCB and constructed spectrograms to visualize any inertial cavitation content. We beamformed 3 out of the 69 bursts of 100 µs of data into an RCB-PAM for 3 foci sonication and 1/69 for one focus sonication. We used these maps to calculate mean $\overrightarrow {{{\varvec{T}}_{{{\varvec{error}}\_{\varvec{PAM}}}} }}$. In this manner, we computed a total of 450 images of RCB maps for 150 s of sonication time for 3 foci BBB opening sonications and 150 maps for one focus BBB opening sonications. Our baseline sonications consisted of 10 maps. Creation of RCB maps for all 10,350 echoes was not tractable due to computational expense. Hence, for $\overrightarrow {{{\varvec{T}}_{{{\varvec{error}}\_{\varvec{PAM}}}} }}$ we used the first three frame for multiple foci and only first frame of single focus BBB opening experiment. To calculate ($\overrightarrow {{{\varvec{T}}_{{{\varvec{error}}\_{\varvec{PAM}}}} }} )$ of our in vivo data, we first found the mean intensity of all 150 BBB opened maps and created a single map for each in vivo trial. We found the pixel with the maximum intensity in the mean map and subtracted it with the intended focus to calculate ($\overrightarrow {{{\varvec{T}}_{{{\varvec{error}}\_{\varvec{PAM}}}} }} )$. We then compared the mean intensity in baseline maps (n = 10) versus BBB opened maps (n = 150) using Wilcoxon rank-sum test, to compare unequal sample sizes, and considered *p* < 0.05 to be statistically significant.

### Gadolinium contrast-enhanced imaging to confirm BBB opening

We anesthetized adult male rats with 2–3% isoflurane (ISO) for induction and 1.5–2% for maintenance during MR imaging. We monitored and maintained respiration and rectal temperature during MRI. Respiration was kept around 60 cycles/min, and a rectal temperature of 37.5 °C was maintained throughout the experiments using a warm-air feedback system (SA Instruments, Stony Brook, NY, USA). We collected MRI data on a Varian DirectDrive™ horizontal 4.7 T magnet using a 38-mm inner diameter transceiver coil (Doty Scientific Inc. Columbia, SC, USA). We acquired T1W images before and after gadolinium injection (Bayer HealthCare Pharmaceuticals, Whippany, NJ, USA) for confirming BBB opening (3D spoiled gradient echo sequence, TR/TE = 9/2.37 ms, flip angle = 7°, number of excitations = 8, matrix size = 128 × 128 × 64, field of view = 32 × 32 × 16 mm^3^, resolution = 0.25 × 0.25 × 0.25 mm^3^, acquisition time of 9 min 50 s). To calculate BBBO opening volume, we took an ROI encompassing the BBB opened region. We, then, measured all the pixels with intensity of 2.5 times the standard deviation of the background and multiplied this number of voxels with the voxel volume. To visualize BBB opening, we identified the MRI plane with the greatest BBB opening surface area in the ROI above, normalized this area inside the ROI to the maximum, and overlayed the map onto the T1w images.

### MRI to ultrasound image registration

We selected points surrounding the ventricles, arteries and cortex that are present in both power Doppler image and MRI image and performed a point registration of the points in the Gd subtracted MRI image to the points in the power Doppler image (Fig. 5b) using MATLAB’s fitgeotrans function of MATLAB after selecting groups of points in the MRI image and power Doppler image. Finally, we used imwarp function to transform MRI image according to geometric transformation in parameter tform. Point registered MRI images were used to calculate target error vector of opening BBB $\overrightarrow {{{\varvec{T}}_{{{\varvec{error}}\_{\varvec{BBB}}}} }}$ in Eq. 10, which is the difference between pixel of highest intensity of BBB opened region in the MRI image ($pixel_{MRI} = \left[ {x_{MRI} ,y_{MRI} } \right])$ and location of the pixel that was clicked on the power Doppler image ($pixel_{PD} = \left[ {x_{PD} ,y_{PD} } \right]$) to open BBB. This was possible because after registration, both MRI image and power Doppler images were in the same coordinate system.

10 $$\overrightarrow {{{\varvec{T}}_{{{\varvec{error}}\_{\varvec{BBB}}}} }} = \sqrt {(x_{MRI} - x_{PD} )^{2} + (y_{MRI} - y_{PD} )^{2} }$$

## Supplementary Information

Supplementary Information 1.

Supplementary Information 2.

Supplementary Information 3.

Supplementary Information 4.

## Data Availability

The dataset generated and used in this study will be from the corresponding author upon request.
