# Supplementary material for: Guiding and monitoring focused ultrasound mediated blood–brain barrier opening in rats using power Doppler imaging and passive acoustic mapping
Source: Sci Rep. 2022 Aug 30;12:14758. doi: 10.1038/s41598-022-18328-z (PMC9427847; doi:10.1038/s41598-022-18328-z)
Supplement: Supplementary file 1 — Supplementary Information 1. [file 41598_2022_18328_MOESM1_ESM.pdf]

a) <u>B-Mode images of hydrophone tip</u>

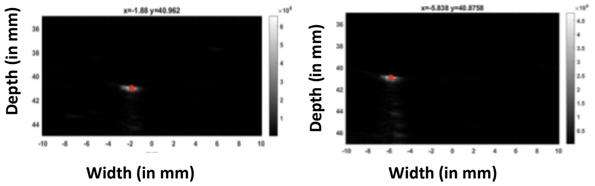

b) White band at top of the image shows the active aperture of imaging transducer for receiving passive echoes

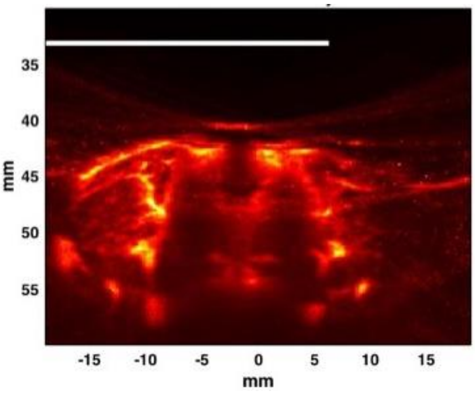

Supplementary fig S1: Hydrophone B-Mode images and active elements of our imaging transducer. a) Our imaging transducer recorded images of hydrophone tip. We processed these images offline to get positions of the hydrophone tip. b) All 192 elements of our imaging transducer was used to make power Doppler image but only 128 elements out of 192 elements of our imaging transducer, presented by the white band, was used for capturing receive echoes for PAM.
