# Supplementary material for: Guiding and monitoring focused ultrasound mediated blood–brain barrier opening in rats using power Doppler imaging and passive acoustic mapping
Source: Sci Rep. 2022 Aug 30;12:14758. doi: 10.1038/s41598-022-18328-z (PMC9427847; doi:10.1038/s41598-022-18328-z)
Supplement: Supplementary file 2 — Supplementary Information 2. [file 41598_2022_18328_MOESM2_ESM.pdf]

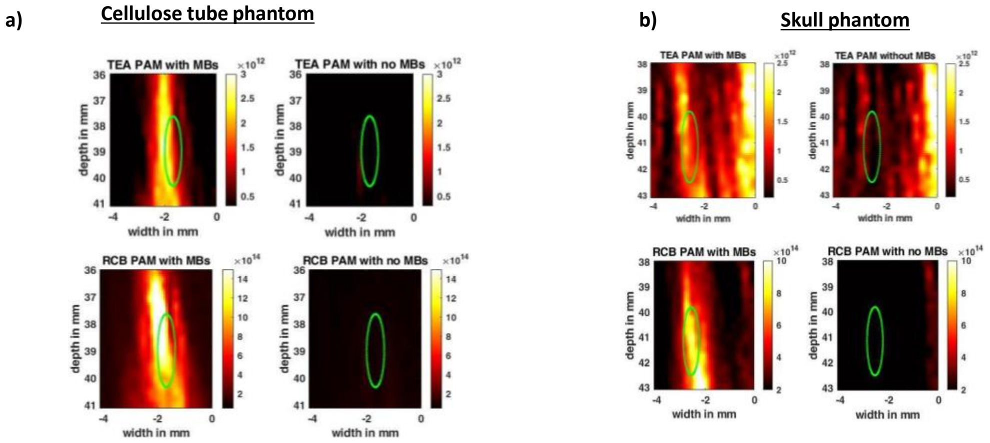

Supplementary fig S2: Comparison of TEA PAM with RCB PAM. a) Mean RCB-PAM and TEA-PAM for entire FUS pulse can create passive acoustic maps at the targeted location in cellulose tube phantom. b) In skull phantom case, only RCB-PAM can create accurate passive acoustic maps of regions undergoing cavitation for entire FUS pulse. TEA-PAM cannot resolve regions under cavitation with same accuracy as RCB-PAM. TEA-PAM shows off-target regions to have more spectral energy than focal regions.
