# Supplementary material for: Guiding and monitoring focused ultrasound mediated blood–brain barrier opening in rats using power Doppler imaging and passive acoustic mapping
Source: Sci Rep. 2022 Aug 30;12:14758. doi: 10.1038/s41598-022-18328-z (PMC9427847; doi:10.1038/s41598-022-18328-z)
Supplement: Supplementary file 3 — Supplementary Information 3. [file 41598_2022_18328_MOESM3_ESM.pdf]

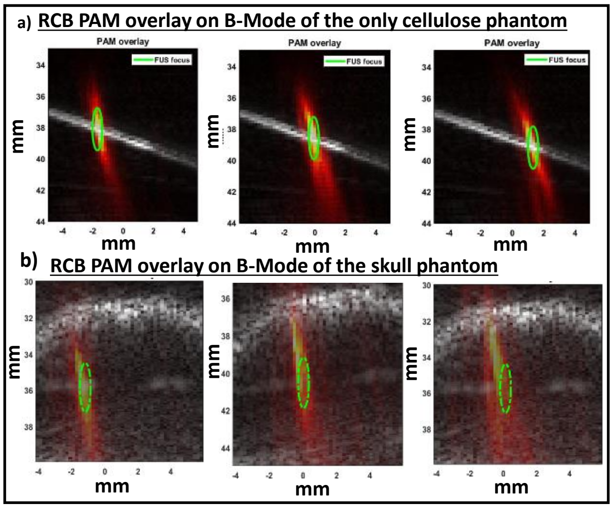

Supplementary fig S3: RCB PAM overlay on B-Mode images are near the intended FUS focus. a) RCB PAM could localize steered FUS focus and majority of PAM signal was inside FUS focus. b) RCB PAM could localize FUS focus in skull phantom set up as well. In 2/3 cases, majority of the PAM signal was inside FUS focus.
