# Supplementary material for: Guiding and monitoring focused ultrasound mediated blood–brain barrier opening in rats using power Doppler imaging and passive acoustic mapping
Source: Sci Rep. 2022 Aug 30;12:14758. doi: 10.1038/s41598-022-18328-z (PMC9427847; doi:10.1038/s41598-022-18328-z)
Supplement: Supplementary file 4 — Supplementary Information 4. [file 41598_2022_18328_MOESM4_ESM.pdf]

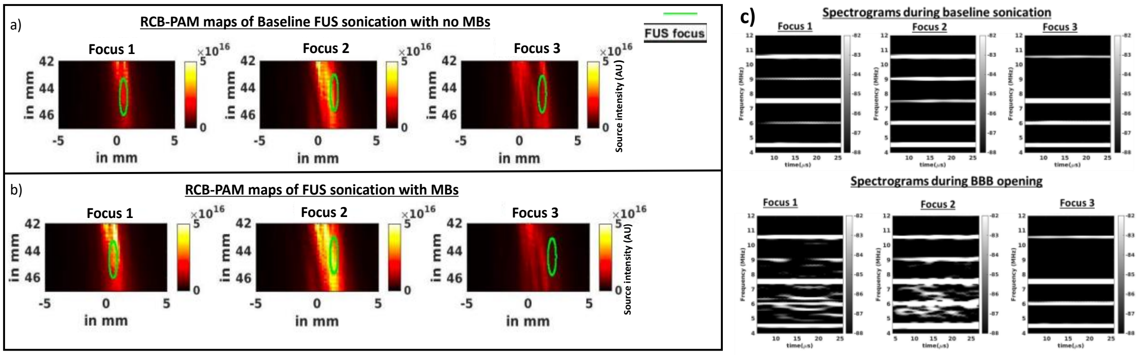

Supplementary figure S4: RCB-PAM can localize cavitation signals in an in vivo rat brain with higher source intensity in presence of MBs than in absence. a) RCB-PAM maps of of focus 1, focus 2 and focus 3 of first sonication of baseline show low magnitude intensities. Focus 2 shows some bright pixel but this pixel is close to the skull and could be attributed to skull reflection. The magnitude of the brightest pixels in focus 1 is $3.19e^{16}$, in focus 2 is $4.6e^{16}$, and in focus 3 is $2e^{16}$. b) RCB PAM image of first FUS sonication with microbubbles show high intensity PAM maps. There is high intensity activity near the intended focus for foci 1 and 2. The PAM maps for focus 3 look similar to the baseline focus 3. The magnitude of the brightest pixels in focus 1 is $7.6e^{16}$, in focus 2 is $4.9e^{16}$ and in focus 3 is $2.1e^{16}$. The magnitude of the brightest pixel is more than 2 times in RCB-PAM map of focus 1 of FUS sonications with MBs than with no MBs. The value of brightest pixel in focus 2 in RCB-PAM maps with MBs is larger than without MBs but not as large as focus 1. However, the regions with higher energy is greater in RCB-PAM maps of focus 2 with MBs than without MBs. In RCB-PAM maps of focus 3 for both with and without MBs, these values are comparable. We reconstructed first 3/69 pulses for each 150 sonications. c) An individual spectrograms of last 30us first FUS sonication shows that there is more harmonic, ultraharmonics and inertial content in presence of MBs than in absence of MBs. We reconstructed all 69 spectrograms, comprising 6.9ms, of each sonication performed at 1Hz to compute cavitation dosage for entire 150s of treatment.
